# A Sub-1 Hz Resonance Frequency Resonator Enabled by Multi-Step Tuning for Micro-Seismometer

**DOI:** 10.3390/mi13010063

**Published:** 2021-12-30

**Authors:** Jun Wu, Hideyuki Maekoba, Arnaud Parent, Tamio Ikehashi

**Affiliations:** 1 Graduate School of Information Production and Systems, Waseda University, Kitakyushu-shi 808-0135, Japan; woojun@fuji.waseda.jp; 2 Coventor, A Lam Research Company, Fremont, CA 94538, USA; Hideyuki.Maekoba@lamresearch.com (H.M.); aparent@coventor.com (A.P.)

**Keywords:** MEMS, seismometer, resonance frequency, force-balanced, electrical tuning

## Abstract

We propose a sub-1 Hz resonance frequency MEMS resonator that can be used for seismometers. The low resonance frequency is achieved by an electrically tunable spring with an ultra-small spring constant. Generally, it is difficult to electrically fine-tune the resonance frequency at a near-zero spring constant because the frequency shift per voltage will diverge at the limit of zero spring constant. To circumvent this issue, we propose a multi-step electrical tuning method. We show by simulations that the resonance frequency can be tuned by 0.008 Hz/mV even in the sub-1 Hz region. The small spring constant, however, reduces the shock robustness and dynamic range of the seismometer. To prevent this, we employ a force-balanced method in which the mass displacement is nulled by the feedback force. We show that the displacement can be obtained from the voltage that generates the feedback force.

## 1. Introduction

A seismometer is a sensor that detects ground motions [1]. Mechanical seismometers adopt a mass-spring-damper system with a low resonance frequency so that the proof mass behaves as a fixed point relative to the ground. To achieve a resonance frequency of about 1 Hz, a kg-level heavy proof mass is usually used [2]. But the large mass and size is not suited for IoT applications that require compact seismometers [3].

Recently, mainly in the field of gravimeters, low resonance frequency MEMS structures are reported, as summarized in Table 1. In the case of Middlemiss’ gravimeter, which has a similar structure to a seismometer, the resonance frequency of 2.3 Hz was achieved by an ultra-small spring constant composed of two kinds of mechanical spring, one of them having a negative spring constant [4].

This paper aims to extend the approach to make a MEMS resonator with sub-1 Hz resonance frequency. Our final goal is to make a compact MEMS seismometer that can be used as an IoT sensor node. To achieve the small resonance frequency, we introduce an electrically tunable negative spring constant enabled by parallel plate electrodes. Compared with the conventional method, electrical tunability has three advantages: (i) cancellation of process variation of spring constant, (ii) compensation of temperature dependence of spring constant, and (iii) yield improvement during fabrication and handling. The last point is attained by the larger spring constant of the power-off state, i.e., when no voltage is applied to the electrodes.

Electrical tuning of a resonance frequency is a well-known method [10,11]. However, making near zero resonance frequency is by no means easy, since the frequency shift ∆*f* induced by a voltage shift ∆*V* will become very large when the spring constant k becomes small. This is due to the fact that $df/dV\propto 1/\sqrt{k}$. To resolve this issue, we introduce a multi-step electrical tuning method. We show that ∆*f* can be made small enough even for the small spring constant.

The small spring constant, however, brings about some drawbacks. One is the re-duction of shock robustness and the other is the decrease in dynamic range when applied to a seismometer. To circumvent these issues, we employ a force-balanced system. Namely, the displacement of the mass is sensed, and then a feedback force is generated based on the result, to cancel the displacement. Force-balanced methods have already been adopted in seismometers [12], but our system enables minimizing the size, since it only uses electrostatic forces.

## 2. Design

### 2.1. Electrically Tunable Spring

In a spring-mass system of spring constant *k* and mass *m*, the resonance frequency is given by

(1) $$f=\frac{1}{2\pi }\sqrt{\frac{k}{m}}.$$

**Figure 1:**
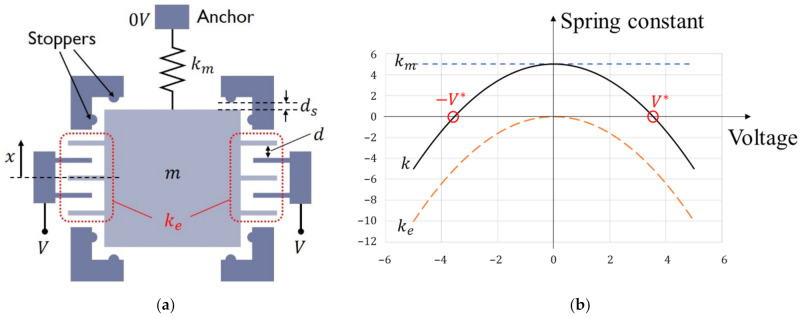
The basic principle of composite springs: (a) Schematic of seismometer structure. Total spring k is given by a sum of mechanical spring $k_{m}$ and electrical spring $k_{e}\left(\propto V^{2}\right).$ Stoppers with gap $d_{s}$ which is smaller than the electrode gap d, can prevent electrical short. (b) Behaviors of total spring $k=k_{m}+k_{e}$.

This formula indicates that small resonance frequency can be attained by small *k* even for small *m*. To account for process variations and robustness during the fabrication, it would be advantageous to attain the small k by electrical tuning [13]. A schematic of this electrically tunable spring is shown in Figure 1a. The total spring k is composed of mechanical spring $k_{m}$ and parallel plate electrodes. Due to the symmetry of the electrode structure, attractive forces occur at both sides of the fixed electrodes. Noting that the force magnitude is proportional to $V^{2}$, the electrostatic force is written as $F_{e}=\alpha V^{2}x$, with α being a constant. This implies that the electrostatic force can be regarded as a spring force with a negative spring constant, namely, $F_{e}=-k_{e}x$ with $k_{e}=-\alpha V^{2}\left(<0\right).$ The total spring constant thus becomes the sum of the two springs, $k=k_{m}+k_{e}$, and it can be tuned by the voltage V. Electrical short between the electrodes can be avoided by introducing stoppers, as in Figure 1a. As shown in Figure 1b, there are two points that can make zero *k*. The resonance frequency of this mechanical system shown in Figure 1a is

(2) $$f=\frac{1}{2\pi }\sqrt{\frac{k_{m}-\alpha V^{2}}{m}}.$$

If the voltage is changed as V→V+∆V, the frequency will change as

(3) $$f^{\prime }=f+\frac{df}{dV}∆V,$$

where

(4) $$\frac{df}{dV}=-\frac{\alpha V}{2\pi \sqrt{m\left(k_{m}-\alpha V^{2}\right)}}\approx -\frac{k_{m}}{k}\cdot \frac{f}{V}.$$

This expression implies that as k becomes smaller, df/dV becomes larger, and it will become more difficult to fine-tune the frequency. For example, to attain a resonance frequency of 1 Hz with a 10 mg proof mass, the spring constant k should be as small as $4\times 10^{-4\;}N/m$. If we further assume $k_{m}=4\;N/m$ and V=10 V, we obtain $\left|df/dV\right|=1000\;\mathrm{Hz}/V$. Thus, a small voltage fluctuation will lead to a large shift in the resonance frequency. This is the difficulty that needs to be resolved in order to attain f<1 Hz.

### 2.2. Multi-Step Electrical Tuning Method

To overcome the difficulty of fine-tuning, we employ a multi-step tuning method. In this method, we introduce three kinds of parallel plate electrodes to which different voltages can be applied. The frequency is then expressed as

(5) $$f=\frac{1}{2\pi }\sqrt{\frac{k_{m}-\alpha_{1}V_{1}^{2}-\alpha_{2}V_{2}^{2}-\alpha_{3}V_{3}^{2}}{m}}\;,\;$$

where $V_{i}$’s are three independent voltages and $\alpha_{i}$’s are coefficients determined by electrode size. In terms of vacuum permittivity $\varepsilon_{0}$, electrode gap d and overlap area of *i*-th electrode $A_{i}\;\left(i=1,2,3\right)$, the coefficient $\alpha_{i}$ is written as $\alpha_{i}=2\varepsilon_{0}A_{i}/d^{3}$. The frequency change Δf induced by the voltage change $\Delta V_{1},\;\;\Delta V_{2},\;\;\Delta V_{3}$ is written as

(6) $$\Delta f=\frac{\partial f}{\partial V_{1}}\Delta V_{1}+\frac{\partial f}{\partial V_{2}}\Delta V_{2}+\frac{\partial f}{\partial V_{3}}\Delta V_{3}\;,$$

where

(7) $$\frac{\partial f}{\partial V_{i}}=-\frac{\alpha_{i}V_{i}}{2\pi \sqrt{m\left(k_{m}-\alpha_{1}V_{1}^{2}-\alpha_{2}V_{2}^{2}-\alpha_{3}V_{3}^{2}\right)}}.$$

If we set a condition $\alpha_{1}\gg \alpha_{2}\gg \alpha_{3}$ which can be attained by changing the electrode area $A_{i}$, we get

(8) $$\left|\frac{\partial f}{\partial V_{1}}\right|\gg \left|\frac{\partial f}{\partial V_{2}}\right|\gg \left|\frac{\partial f}{\partial V_{3}}\right|.$$

**Figure 2:**
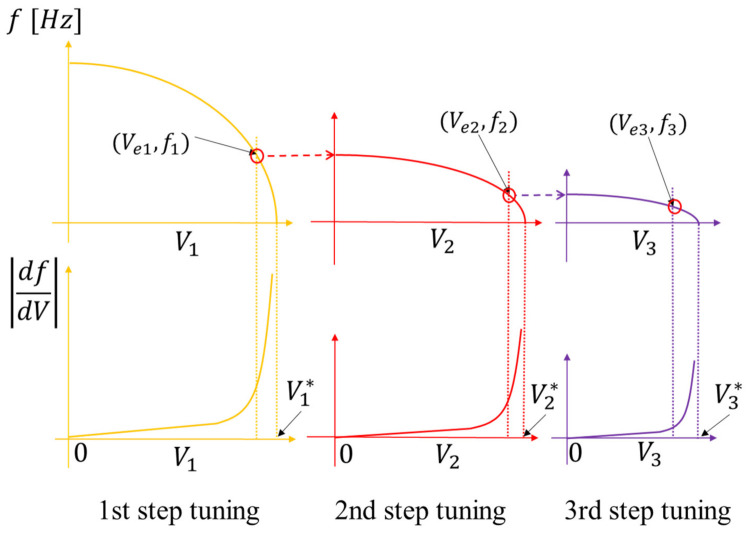
The basic principle of multi-step electrical tuning. In the 1st, 2nd, 3rd step tunings, frequencies will become 0 at $V_{1}^{*},\;\;V_{2}^{*},\;V_{3}^{*}$_,_ respectively, and $\left|df/dV\right|$ goes to infinity at those voltages. Voltages $V_{e1},\;\;V_{e2},\;\;V_{e3}$ are voltages slightly smaller than $V_{1}^{*},\;\;V_{2}^{*},\;\;V_{3}^{*}$.

**Figure 3:**
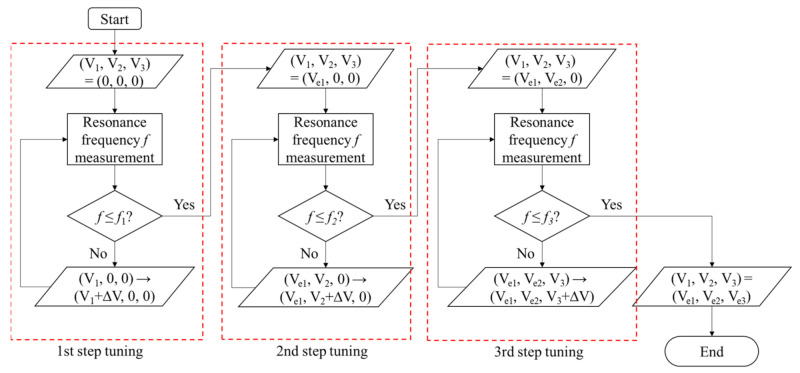
The flow chart of multi-step electrical tuning. Voltages $V_{1},\;\;V_{2},\;V_{3}$ are the voltages applied to 1st, 2nd, 3rd step tuning electrodes, respectively. And $f_{1},\;\;f_{2},\;f_{3}$ are the target resonance frequencies of each step. We set $f_{1}=8.0\;\mathrm{Hz},\;\;f_{2}=1.9\;\mathrm{Hz},\;\;f_{3}=0.4\;\mathrm{Hz}$ for our resonator.

This suggests that we can fine-tune the frequency by optimizing the voltages $V_{1},\;V_{2},V_{3}$ in this order. A practical method of multi-step tuning is depicted in Figure 2 and the tuning flow is shown in Figure 3. In this method, we stop the tuning of the first step before the slope $\left|df/dV\right|$ goes to infinity and start tuning of the second step. Similarly, we stop the second tuning before the slope goes to infinity and move on to the third step. By repeating this procedure, a small resonance frequency can be attained with reasonably small $\left|df/dV\right|$, which implies availability of fine frequency tuning. The number of tuning steps can be increased depending on the target frequency.

### 2.3. Seismometer Structure

**Figure 4:**
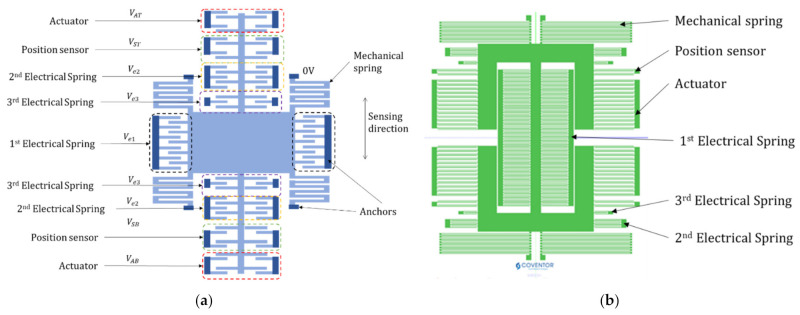
(**a**) Schematic of seismometer structure. (**b**) Top view of the seismometer layout used in a FEM simulator *MEMS*+.

The mechanical structure of our seismometer is shown in Figure 4. This structure can be fabricated by applying deep reactive ion etching (DRIE) to a silicon layer. Three sets of electrical springs, namely 1st, 2nd and 3rd electrical springs, are formed by symmetrical parallel plate electrodes. We also have position sensors and actuators composed of asymmetric parallel plate electrodes, which are used in the force-balanced system. The main dimensions are provided in Table 2.

### 2.4. Fabrication

**Figure 5:**
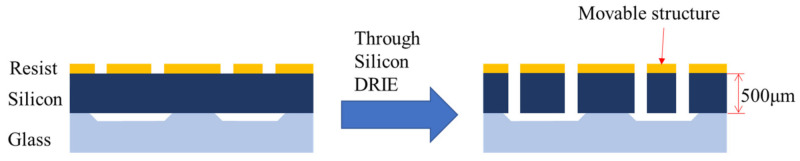
Schematic fabrication process. Through silicon DRIE is applied to the silicon layer of the bonded wafer. The minimum gap of the silicon layer is 25 μm to attain an aspect ratio of 20.

Figure 5 shows a schematic fabrication process of our device. Firstly, recess patterns were made on a glass wafer. Then the wafer is anodic bonded to a silicon wafer. Finally, through silicon DRIE is done to make the movable portion.

### 2.5. Demonstration of Multi-Step Tuning

We are now in a position to demonstrate the fine-tuning procedure for the seismometer. To simulate the device performance, Coventor’s multi-physics simulation tool, *MEMS*+^®^ ,is employed [14]. Past studies proved *MEMS*+ accurately predicts experimental electrostatic softening for manufactured gyroscopes [11,15]. The top view of the *MEMS*+ simulated model is shown in Figure 4b. Firstly, when the three voltages $V_{1},\;\;V_{2},\;V_{3}$ are set to zero, the resonance frequency of the sensing direction was 90.3 Hz. Resonance frequencies of other modes are much higher than this value due to larger spring constants. 

**Figure 6:**
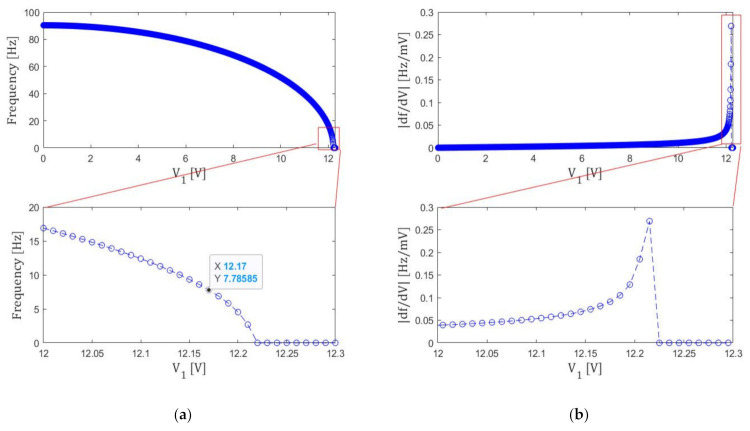
(a) Voltage $V_{1}$ dependence of the resonance frequency f. (b) Its derivative $\left|\partial f/\partial V_{1}\right|$.

In the first step tuning, voltage $V_{1}$ is optimized with $V_{2}=V_{3}=0$. As shown in Figure 6, the resonance frequency f will decrease with increasing $V_{1}$. If $V_{1}$ is changed with the 10 mV step, the resonance frequency f becomes minimum when $V_{1}$ is 12.220 V$\;\equiv \;V_{1}^{*}$, as shown in the inserted figure. However, as indicated in Figure 6b, the frequency slope $\left|\partial f/\partial V_{1}\right|$ will become infinite as $V_{1}$ approaches to $V_{1}^{*}$. This indicates that fine frequency tuning is not possible at the vicinity of the $V_{1}$ that minimizes f.

Based on the flow chart of Figure 3, we stop the 1st step tuning when the resonance frequency becomes smaller than 8.0 Hz. As a result, we find that $V_{1}$ becomes 12.17 V, which is defined as $V_{e1}$. At this $V_{e1}$, frequency resolution can be made smaller than 0.1 Hz/mV. The resonance frequency at this voltage is 7.8 Hz and the spring constant is 0.157 N/m.

**Figure 7:**
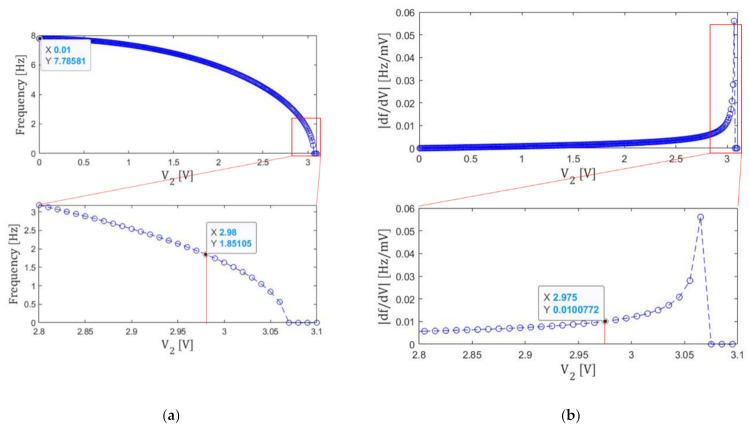
(a) Voltage $V_{2}$ dependence of the resonance frequency f. (b) Its derivative $\left|\partial f/\partial V_{2}\right|$.

We apply the second step tuning to this state. The results are shown in Figure 7. The 2nd step tuning is done until the resonance frequency becomes smaller than 1.9 Hz. Then we find $V_{2}=2.98\;V\equiv V_{e2}$. Though the resonance frequency can be made smaller than 1 Hz in this step, we go to the next step to stabilize the resonance frequency against voltage fluctuations.

At this $V_{e2}$, due to the smaller electrode area, the slope $\left|\partial f/\partial V_{2}\right|$ can be made smaller than 0.01 Hz/mV. The resonance frequency after this step is 1.85 Hz.

**Figure 8:**
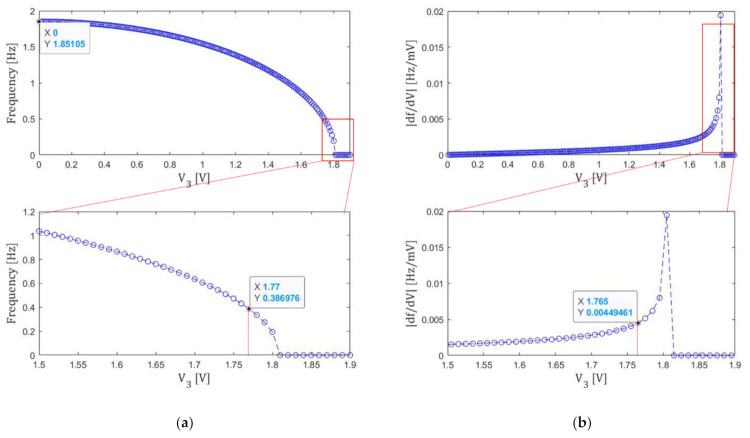
(a) Voltage $V_{3}$ dependence of the resonance frequency f. (b) Its derivative $\left|\partial f/\partial V_{3}\right|\;$.

Finally, 3rd step tuning is applied to this state. The results are shown in Figure 8. The 3rd step tuning is done until the resonance frequency becomes smaller than 0.4 Hz. Then we find $V_{3}=1.77\;V\equiv V_{e3}$. The slope $\left|\partial f/\partial V_{3}\right|$ can now become smaller than 0.008 Hz/mV.

To summarize, we have set $V_{e1}=12.17\;V$, $V_{e2}=2.98\;V$, and $V_{e3}=1.77\;V$. The resulting resonance frequency was found to be 0.39 Hz. Note that the size increase caused by the second and the third tuning is small since their electrode areas are far smaller than the first one.

### 2.6. Force-Balance System

**Figure 9:**
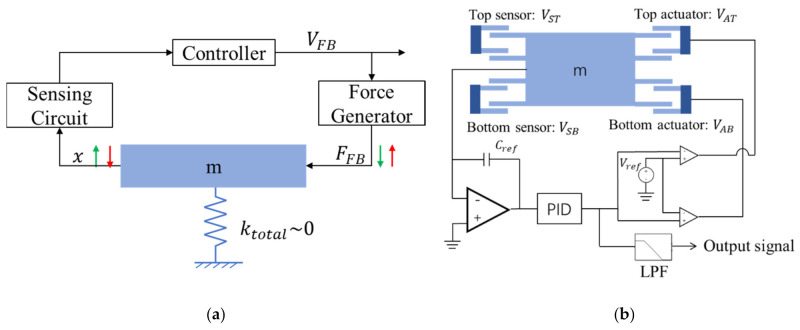
(**a**) Basic concept of force-balance method. (**b**) Circuit block diagram of seismometer.

The small spring constant we have achieved has an issue with respect to the shock immunity and dynamic range of the sensor. To circumvent this, we introduce a force-balanced approach. The concept of the force-balanced system is shown in Figure 9a. When a displacement occurs due to an external force, the position sensor detects the displacement, and a force will be applied to nullify the displacement. The displacement can be known from the voltage required to cancel the displacement.

This kind of force-balanced system is already adopted in seismometers. The conventional system, however, uses magnetic force that calls for many components and bulky coil [12]. On the other hand, the present system can be made more compact since it only uses electrostatic force, and therefore the whole mechanical system can be embedded in a single chip.

A schematic block diagram of our force-balanced system is shown in Figure 9b. A pair of asymmetrical gap electrodes are used for the position sensor and actuators. To minimize the offset and overshooting/undershooting of the output voltage $V_{FB}$, we introduce a PID controller. Based on the voltage $V_{FB}$, upward and downward forces are generated by the top and bottom actuators.

**Figure 10:**
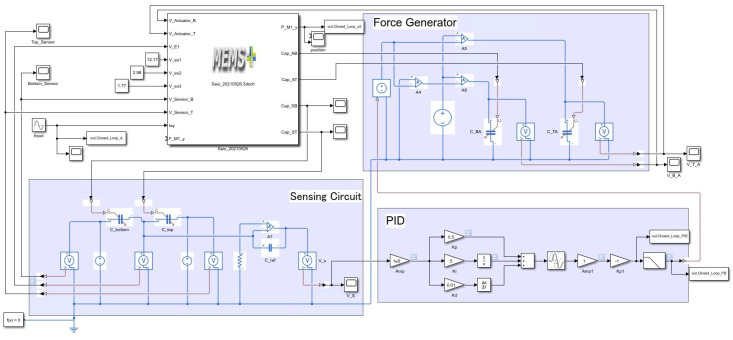
Block diagram of the multi-domain simulation.

**Figure 11:**
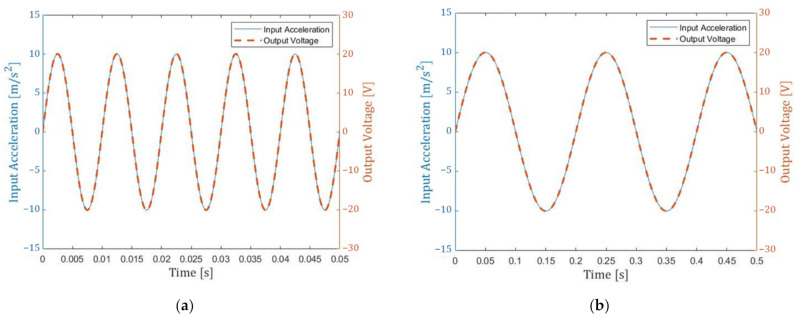
Simulation results of different acceleration input frequencies (**a**) 100 Hz (maximum frequency); (**b**) 5 Hz (typical frequency). The solid line corresponds to the input acceleration, and the dashed line corresponds to the output voltage. Matching of the two curves implies that the system can respond to the input accelerations. Results for lower frequencies are not shown because catching up to the input will become much easier.

**Figure 12:**
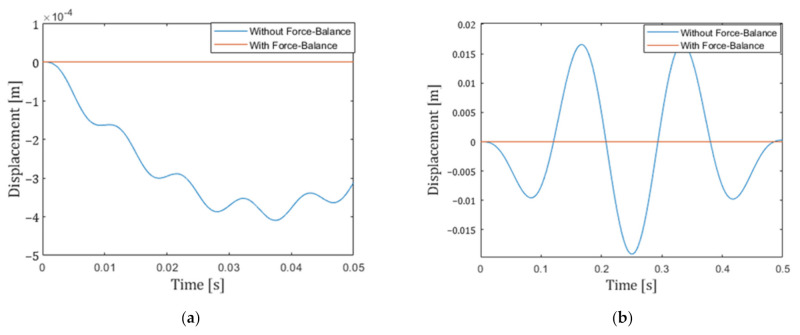
Simulation results of different acceleration input frequencies (**a**) 100 Hz; (**b**) 5 Hz. The *x*-axis is the time; the *y*-axis is displacements of the proof mass in different cases. The displacement becomes smaller for the higher frequency due to the small resonance frequency of 0.39 Hz.

We have also undertaken multi-domain simulation of the device coupled to the control system. Here, the MEMS+ model is employed directly in MathWorks Simulink together with other system blocks to model the control loop. The block diagram of the total system is shown in Figure 10. The target frequency range of our seismometer is 0.01 Hz to 100 Hz. To confirm the response of our system, we carried out simulations at the typical frequency 5 Hz and the maximum frequency 100 Hz. The simulation results are shown in Figure 11 and Figure 12. The results show that the input acceleration is correctly reflected in the output voltage and at the same time, the displacement is canceled. The cancellation of the displacement implies that it can also eliminate nonlinearities that occur at large displacements. The nonlinearity originates from both mechanical spring and electrostatic forces.

The open-loop sensitivity, namely the sensitivity when the force-balanced system is turned off, is given by

(9) $$\frac{∆C}{∆g}=\frac{∆C}{∆x}\cdot \frac{∆x}{∆g}=\frac{∆C}{∆x}\cdot \frac{1}{{\left(2\pi f\right)}^{2}},$$

where *f* is the resonance frequency. Using the simulated value for ∆*C/*∆*x* and 0.39 Hz for *f*, we find that the sensitivity becomes 13.9 nF/(m/s^2^). When the force-balance system is turned on, the displacement is nulled, and the sensitivity becomes 2.0 V/(m/s^2^).

## 3. Conclusions

In this paper, a design methodology of a resonator with ultra-small resonance frequency is presented. Using the proposed three-step electrical tuning, the total spring constant can be easily tuned, and the resonance frequency of the resonator can be tuned to 0.39 Hz and at 0.008 Hz/mV level. A force-balance method that can attain shock robustness and dynamic range enhancement is also proposed and verified by simulation. The primary goal of this study is to attain a MEMS seismometer. However, the design scheme presented here can also be applied to other sensors that call for ultra-small resonance frequencies, such as gravimeters and vibration sensors.

## Figures and Tables

**Table 1:** Comparison of the resonance frequencies of conventional works and this work.

Device	Proof Mass	Resonance Frequency
Middlemiss, R. [4]	13.64 mg	2.3 Hz
Tang, S. [5]	460 mg	3.1 Hz
Pike, W. T. [6,7]	400 mg	6 Hz
Wu, W. [8]	310 mg	14 Hz
Krishnamoort, U. [9]	33.6 mg	36 Hz
This work	24 mg	0.39 Hz

**Table 2:** Dimensions of the seismometer.

Properties	Value	Unit
Si layer thickness	500	μm
Proof mass weight	24	mg
1st electrical spring finger length	1000	μm
1st electrical spring finger number	2 × 50	-
2nd electrical spring finger length	1000	μm
2nd electrical spring finger number	2 × 6	-
3rd electrical spring finger length	500	μm
Position sensor finger length	2 × 2	-
Position sensor finger number	1500	μm
Actuator finger length	2 × 2 × 2	-
Actuator finger number	1500	μm
Minimum gap of electrodes	2 × 2 × 13	-
